# Childhood Experiences and Psychological Distress: Can Benevolent Childhood Experiences Counteract the Negative Effects of Adverse Childhood Experiences?

**DOI:** 10.3389/fpsyg.2022.800871

**Published:** 2022-02-25

**Authors:** Hao Hou, Caochen Zhang, Jie Tang, Jingjing Wang, Jiaqi Xu, Qin Zhou, Wenjun Yan, Xiuyin Gao, Wei Wang

**Affiliations:** ^1^Department of Community and Health Education, School of Public Health, Xuzhou Medical University, Xuzhou, China; ^2^School of Medical Technology, Xuzhou Medical University, Xuzhou, China; ^3^The Affiliated Xuzhou Oriental Hospital of Xuzhou Medical University, Xuzhou, China; ^4^Center for Medical Statistics and Data Analysis, Xuzhou Medical University, Xuzhou, China; ^5^Key Laboratory of Human Genetics and Environmental Medicine, Xuzhou Medical University, Xuzhou, China

**Keywords:** benevolent childhood experiences, adverse childhood experiences, psychological distress, undergraduates, China

## Abstract

**Background:**

Childhood experiences can exert a huge impact on adult psychological conditions. Previous studies have confirmed the effects of adverse childhood experiences (ACEs) and benevolent childhood experiences (BCEs) on psychological distress (e.g., stress, depression, and suicidal ideation) separately, but few studies explored a combined effect of ACEs and BCEs on psychological distress. The aim of this study was to explore a combined effect of ACEs and BCEs on psychological distress among Chinese undergraduates.

**Methods:**

Participants were undergraduates aged 17–24 years (*N* = 1,816) and completed a self-reported questionnaire. A series of regression analyses were conducted to examine the association between childhood experiences and psychological distress.

**Results:**

A total of 65.7% of undergraduates had BCEs, 27.1% of undergraduates had ACEs, and 12.9% of undergraduates had ACEs and BCEs simultaneously. Logistic regression analysis indicated that undergraduates who experienced high ACEs were more likely to have a high risk of psychological distress [odds ratio (ORs) = 1.46, 1.84, and 3.15 for uncertainty stress, depressive symptoms, and suicidal ideation, respectively], while undergraduates who experienced High BCEs were less likely to have psychological distress (ORs = 0.33, 0.22, and 0.32 for uncertainty stress, depressive symptoms, and suicidal ideation, respectively) compared with Low-Both group. The combined effect of ACEs and BCEs (High-Both group) could also play as a protective factor in uncertainty stress (OR = 0.56) and depressive symptoms (OR = 0.47).

**Conclusion:**

Our findings suggested that ACEs and BCEs could not only predict the psychological distress independently, but also BCEs could counteract the negative effect of ACEs in psychological problems. There is an even greater need to identify and support the victims of ACEs and to increase BCEs in early childhood.

## Introduction

Depression and suicidal ideation are worldwide concerns and they are the most common mental health problems (Bilsen, 2018; Perquier et al., 2021; Pozuelo et al., 2022). Existing studies indicated that 19.6–30.6% of adolescents suffered from depression (Song et al., 2008; Ibrahim et al., 2013; Gao et al., 2020), while 18–26.4% (Baiden et al., 2021; Hu et al., 2021; Mahumud et al., 2021) had suicidal ideation. In recent years, another mental problem, i.e., uncertainty stress, has attracted some attention from scholars (Holland and Wheeler, 2016), because it can arouse worse consequences and influences than life stress and study stress (Wu et al., 2020). Uncertainty stress refers to the stress caused by the condition of being unsure about someone or something (Yang et al., 2017). Since most students are confused about their own life goals and future (Wu et al., 2016; Yang et al., 2018; Peng et al., 2020), they are easily affected by the rapid socioeconomic transition, increased job competition, immature social values, and feelings of social anomie, which make them become a vulnerable population of uncertainty stress (Yang et al., 2019; Peng et al., 2020). Yang et al. (2019) found that 31.1% of Chinese university students suffered from uncertainty stress. All the depressive symptoms, suicidal ideation, and uncertainty stress could arouse negative adult outcomes, which were collectively known as psychological distress (Schneider et al., 2012).

Existing studies have confirmed that psychological distress could be aroused by negative events in the adult stage (Soares et al., 2020). However, except for the events in the adult stage, it is noteworthy that psychological distress is also linked to childhood experiences, e.g., adverse childhood experiences (ACEs) (Clements-Nolle et al., 2018; Bethell et al., 2019; Tsehay et al., 2020; Doom et al., 2021). ACEs represent a series of harmful experiences that occurred before 18 years old (Merrick et al., 2018). In comparison with those who get no exposure to ACEs, people exposed to ACEs have a higher probability of getting physical and mental health problems and even premature mortality (Chung et al., 2008; Hughes et al., 2016). A meta-analysis showed that 22–24.8% of adolescents reported at least one adverse experience (Bellis et al., 2019). Specifically, ACEs have a significant impact on stress, depressive symptoms, and suicidal ideation (Kim, 2017; Ding et al., 2019; Tracy et al., 2019; Karatekin and Ahluwalia, 2020). For instance, ACEs were attributed to 30% cases of anxiety and 40% cases of depression in North America, and more than a quarter of cases of both conditions in Europe (Bellis et al., 2019). In China, adolescents with 3–5 ACEs are over 10 times likely to be depressed and more than 20 times to have suicidal attempts (Jia et al., 2020). Finally, the stress sensitization hypothesis indicated that ACEs hinder the development of brain areas in charge of stress regulations (McLaughlin et al., 2010) which could not only lead to mental health problems but also trigger subsequent new stressors that were not previously present (Aneshensel and Mitchell, 2014).

Contrary to ACEs, benevolent childhood experiences (BCEs) represent the positive experiences before 18 years, which do not depend on higher socioeconomic status in the family of origin. BCEs can not only provide a foundation for creating better family health in adulthood (Daines et al., 2021) but are also linked to various adult mental health outcomes, such as stress, depression (Bethell et al., 2019), forgiveness, family closeness (Crandall et al., 2019), post-traumatic stress disorder (PTSD) (Narayan et al., 2017; Karatzias et al., 2020), loneliness (Doom et al., 2021), and later life cognition (Lee and Schafer, 2021). BCEs were also significantly associated with adulthood insomnia among young adults (Geng et al., 2021). Furthermore, BCEs were associated with ideal cardiovascular health in midlife (Slopen et al., 2017) and showed a better prognosis in patients with personality disorders (Skodol et al., 2007).

Crandall et al. (2019) introduced the Resiliency Theory (Cicchetti, 2016) as a theoretical framework of the relationship between ACEs and BCEs. The Resiliency Theory suggested that multiple systems (e.g., individual, family, neighborhoods, and schools) interact to affect the course of development and that resilience itself is constantly evolving within individuals and systems (Zimmerman, 2013; Crandall et al., 2019), which postulate that positive or protective factors have a direct and independent effect on an outcome separate from a risk factor. Furthermore, these positive factors can neutralize the effect of risk factors on an outcome (Zimmerman, 2013; Crandall et al., 2019). While ACEs have a negative effect on adult health, BCEs will protect health and promote wellness and may even neutralize the effects of ACEs on adult health behaviors and outcomes.

Up to date, few studies explored the effects of co-occurrence of ACEs and BCEs. On the one hand, one opinion maintained that BCEs and ACEs were only modestly negatively associated, underscoring the independence of adverse and positive early experiences (Merrick J.S. et al., 2019; Doom et al., 2021). On the other hand, Bethell considered that BCEs both co-occur with and operate independently from ACEs in their associations with the adult health outcomes (Bethell et al., 2019). BCEs can foster healthy development, overall wellness, and resilience, which develop a way to prevent and moderate ACEs through the promotion of BCEs (Sege and Harper, 2017). Existing studies have proven that BCEs can neutralize the negative impact of ACEs on adult depression, stress (Crandall et al., 2019), prenatal stress and psychopathology (Narayan et al., 2018), and adulthood PTSD symptoms (Narayan et al., 2017). However, when people suffer from ACEs too excessively, the protective effects of BCEs will be decreased (Crandall et al., 2019).

So far, few studies reported the overlap between ACEs and BCEs (Narayan et al., 2018; Bethell et al., 2019), and none of the study explored the combined effects of ACEs and BCEs toward uncertainty stress and suicidal ideation in young adults. The aim of this study was to document a percentage of overlap between ACEs and BCEs and explore the combined effects of ACEs and BCEs toward psychological distress (e.g., uncertainty stress, depressive symptoms, and suicidal ideation) from an innovative perspective. Based on the previous studies and Resiliency Theory, we hypothesized that (1) ACEs would predict worse psychological distress outcomes; (2) BCEs would predict a less risk of experiencing psychological distress; and (3) BCEs would counteract the negative effects caused by ACEs.

## Materials and Methods

### Participants

This is a cross-sectional study involving undergraduate students in China. From March to May 2021, participants were recruited in 25 universities of three cities (i.e., Xuzhou, Nanjing, and Wuhan) in three provinces. A stratified multistage cluster sampling method was used to select the participants. We determined the number of universities in each city by the size of the city, and then we randomly selected universities in each city. The random sampling method was used to select the two classes in each university, and cluster sampling was then used in each class.

Participants were referred to the designated site and completed an anonymous electronic questionnaire by an investigating application (Wenjuanxing), which is based on the most popular social platform (WeChat). No incentive was provided, and every participant has been informed of the right to withdraw from the investigation. We excluded unreliable or nonconforming questionnaires (logic error and answer time less than 600 s) for quality control. A total of 2,022 undergraduate students completed the questionnaire, and 206 participants were excluded. Finally, a total of 1,816 participants were included in this study with an effective response rate of 89.91%.

The Medical Ethics Committee of Xuzhou Medical University has reviewed and approved the study protocol.

### Measures

#### Demographic Characteristics

Demographic characteristics included gender (male/female), age, grades (freshman/sophomore year/junior year/senior year), living expenses (yuan) (≤1,000/1,001–2,000/2,001–3,000/=3,000), only child (yes/no), residence (urban/rural), and sexual orientation (heterosexual/homosexual/bisexuality/others).

#### Adverse Childhood Experiences

The ACEs Scale developed by the Kaiser-CDC was used to assess the ACE that occurred before the age of 18 years (Petruccelli et al., 2019; Senreich et al., 2020). The Chinese version of the scale has been confirmed its good validity and reliability and has been widely used in China (Xiao et al., 2020; Geng et al., 2021). The scale consists of 3 subscales and 10 items, including abuse (e.g., sexual abuse), neglect (e.g., emotional neglect), and family dysfunction (e.g., parental divorce or separation). Each “Yes” response was scored as 1 and a “No” response was scored as 0. The total score (range 0–10) was calculated by the sum of 10 items, with higher scores indicating greater exposure to adverse events. Cronbach’s α of the scale was 0.729 in this study. We stratified the sample by a standard ACEs score developed by previous studies (0 vs. =1) (Bellis et al., 2014, 2019; Kiburi et al., 2018).

#### Benevolent Childhood Experiences

The BCEs were measured by the Chinese version of the BCEs Scale that verified the validity and reliability in previous studies (Narayan et al., 2018; Zhan et al., 2021). The scale includes 10 items of positive childhood experiences occurring between birth and 18 years. The scale assessed the three aspects of BCEs, such as (1) perceived internal and external safety and security (e.g., having beliefs that gave comfort); (2) positive and predictive quality of life (e.g., having a predictable home routine); and (3) relational support (e.g., having an adult who could provide support or advice but not a parent/caregiver). Each “Yes” response was scored as a 1 and a “No” response was scored as a 0, and the Cronbach’s α of the scale was 0.729 in this study. A total score of BCEs was summed by 10 items (range 0–10), and the higher score reflects more positive childhood experiences. We stratified the sample by BCEs score using a mean split (=8 vs. >8) (Crandall et al., 2019).

#### Uncertainty Stress

The Uncertainty Stress Questionnaire (Yang and Huang, 2003; Wu et al., 2020) was conducted to assess the uncertainty stress. The scale consists of 4 items, including current status uncertainty, social change uncertainty, goal uncertainty, and social value uncertainty. The scale was rated on a standard 5-point Likert rating scale from 0 (no stress) and 4 (excessive stress). A total stress score was summed by single-item scores. A higher score indicates a high level of stress. The Cronbach’s α of the scale was 0.951, which is acceptable in this study. Consistent with prior practice, scores exceeding two on each item indicated “high stress” (Wu et al., 2016).

#### Depressive Symptoms

A 10-item questionnaire Center for Epidemiologic Studies Depression Scale (CESD-10) (Salazar-Pousada et al., 2010; Radloff, 2016) was used to assess the depressive symptoms in the past week. The scale was rated on a Likert rating scale from 0 (rarely or none of the time, <1 day) to 3 (all the time, 5–7 days). Item 5 and Item 8 are scored inversely. The total score of the 10-item questionnaire is calculated to assess the depressive symptoms, and the higher score represents the higher depressive symptoms. The Cronbach’s α is acceptable in this study (0.869). Consistent with prior practice, a cutoff score of 10 or more was classified, respectively, as a higher score and signified higher depressive levels (Guo et al., 2021).

#### Suicidal Ideation

Suicidal ideation was assessed by using a single item: “Have you had suicidal ideation in the past month?” (Yes/No).

### Statistical Analyses

Descriptive analysis was used to describe the demographic characteristics and childhood experiences. To further examine the effect of ACEs and BCEs and the combined effect of them, we used cross-tabulations to classify samples into four groups (Schneider et al., 2012). The Chi-square test was to examine the bivariate associations of childhood experiences with demographic characteristics and psychological distress indicators. Binomial logistic regression analysis was performed to examine the relationship between childhood experiences and psychological distress. Demographic characteristics were controlled in the logistic model. Statistical significance was identified by values of *p* < 0.05. All statistical analyses were based on SPSS 25.0 software.

## Results

### Participant Characteristics

As shown in Table 1, the mean age of the participants was 20.08 years (*SD* = 1.17), and the freshmen accounted for 15.31% of all participants. More than two-third (69.49%) of the undergraduates were women, and about half (56.55%) of them lived in urban areas. The prevalent phenomenon reported was having 1,001–2,000 yuan each month for living expenses (79.19%). Moreover, 89.48% of the respondents reported that they are heterosexual, and the others (10.52%) are non-heterosexual (e.g., homosexual, bisexuality, or not sure).

**TABLE 1:** Demographic characteristics of the participants (*n* = 1,816).

Variables	Categories	No. (%)
Age (mean ± SD)		20.08 ± 1.17
Gender	Male	554 (30.51%)
	Female	1,262 (69.49%)
Grades	Freshman	278 (15.31%)
	Sophomore year	829 (45.65%)
	Junior year	666 (36.67%)
	Senior year	43 (2.37%)
Living expenses (yuan)	≤1,000	92 (5.07%)
	1,001–2,000	1,438 (79.19%)
	2,001–3,000	226 (12.44%)
	>3,000	60 (3.30%)
Only child	Yes	906 (49.89%)
	No	910 (50.11%)
Residence	Urban	1,027 (56.55%)
	Rural	789 (43.45%)
Sexual orientation	Heterosexual	1,625 (89.48%)
	Homosexual	40 (2.20%)
	Bisexuality	108 (5.95%)
	Others	43 (2.37%)

### Prevalence and Overlap of Adverse Childhood Experiences and Benevolent Childhood Experiences

Table 2 presents the descriptive statistics of ACEs and BCEs. Overall, 65.7% (*n* = 1,194) of undergraduates had high BCEs, 27.1% (*n* = 492) of undergraduates had high ACEs, and 12.9% (*n* = 235) of undergraduates had high ACEs and high BCEs simultaneously. The sample had an average ACEs score of 0.51 and had an SD of 1.17. BCEs scores in the sample ranged 0–1 with a mean of 8.67 (*SD* = 1.78).

**TABLE 2:** Prevalence of each item of ACEs and BCEs.

Items	No (%)	Yes (%)
**BCEs**		
(1) Having at least one safe caregiver	103 (5.67)	1,713 (94.33)
(2) Having at least one good friend	38 (2.09)	1,778 (97.91)
(3) Having beliefs that gave comfort	115 (6.33)	1,701 (93.67)
(4) Enjoying school	347 (19.11)	1,469 (80.89)
(5) Having at least one teacher who cared	497 (27.37)	1,319 (72.63)
(6) Having good neighbors	309 (17.02)	1,507 (82.98)
(7) Having an adult (not a parent/caregiver) who could provide support or advice	229 (12.61)	1,587 (87.39)
(8) Having opportunities to have a good time	55 (3.03)	1,761 (96.97)
(9) Having a positive self-concept	383 (21.09)	1,433 (78.91)
(10) Having a predictable home routine	335 (18.45)	1,481 (81.55)
		*M* = 8.67
		*SD* = 1.78
**ACEs**		
(1) Emotional abuse	1,558 (85.79)	258 (14.21)
(2) Physical abuse	1,712 (94.27)	104 (5.73)
(3) Sexual abuse	1,745 (96.09)	71 (3.91)
(4) Emotional neglect	1,668 (91.85)	148 (8.15)
(5) Physical neglect	1,787 (98.4)	29 (1.6)
(6) Parental separation/divorce	1,663 (91.57)	153 (8.43)
(7) Seeing mother abused	1,771 (97.52)	45 (2.48)
(8) Household substance use	1,797 (98.95)	19 (1.05)
(9) Household mental illness	1,750 (96.37)	66 (3.63)
(10) Household members who are incarcerated	1,770 (97.47)	46 (2.53)
		*M* = 0.51
		*SD* = 1.17

The prevalence of each item of childhood experiences is presented in Table 2. With regard to BCEs, a lack of school-related BCEs was the most prevalent in the investigation, namely, about 27.37% of participants mentioned that they did not have at least one teacher who cared and about 19.11% of participants mentioned that they did not enjoy school. Regarding ACEs, the prevalence of items was more obvious: about 14.21% of undergraduates had experienced emotional abuse in their childhood, such as curses, insults, or derogations.

### Participant Characteristics by Childhood Experiences

To further examine the effect of BCEs, ACEs, and co-occurrence of them, we divided all participants into the following 4 groups (Table 3): (1) 52.8% of participants were classified as High-BCEs group who had experienced high BCEs and low ACEs; (2) 14.2% of participants were classified as High-ACEs group who had experienced high ACEs and low BCEs; (3) 20.1% of participants were classified as Low-Both group which meant that participants experienced low BCEs and ACEs; and (4) 12.9% of participants were assigned to High-Both group who had experienced high BCEs and ACEs simultaneously.

**TABLE 3:** Demographic characteristics correlates of childhood experiences.

Characteristics	Low-Both	High-BCEs	High-ACEs	High-Both	χ^2^	*P*
**Gender**						
Male	111 (20.04)	310 (55.96)	62 (11.19)	71 (12.82)	6.445	0.092
Female	254 (20.13)	649 (51.43)	195 (15.45)	164 (13.00)		
**Grades**						
Freshman	53 (19.06)	140 (50.36)	35 (12.59)	50 (17.99)	18.261	0.032
Sophomore year	182 (21.95)	423 (51.03)	127 (15.32)	97 (11.70)		
Junior year	123 (18.47)	372 (55.86)	85 (12.76)	86 (12.91)		
Senior year	7 (16.28)	24 (55.81)	10 (23.26)	2 (4.65)		
**Living expenses**						
<1,000	16 (17.39)	45 (48.91)	17 (18.48)	14 (15.22)	6.805	0.657
1,001–2,000	296 (20.58)	757 (52.64)	206 (14.33)	179 (12.45)		
2,001–3,000	45 (19.91)	123 (54.42)	27 (11.95)	31 (13.72)		
>3,000	8 (13.33)	34 (56.67)	7 (11.67)	11 (18.33)		
**Only child**						
Yes	166 (18.32)	495 (54.64)	123 (13.58)	122 (13.47)	4.792	0.188
No	199 (21.87)	464 (50.99)	134 (14.73)	113 (12.42)		
**Residence**						
Urban	200 (19.47)	538 (52.39)	147 (14.31)	142 (13.83)	2.017	0.569
Rural	165 (20.91)	421 (53.36)	110 (13.94)	93 (11.79)		
**Sexual orientation**						
Heterosexual	323 (19.88)	880 (54.15)	219 (13.48)	203 (12.49)	30.131	<0.001
Homosexual	9 (22.50)	22 (55.00)	4 (10.00)	5 (12.50)		
Bisexuality	24 (22.22)	34 (31.48)	25 (23.15)	25 (23.15)		
Other	9 (20.93)	23 (53.49)	9 (20.93)	2 (4.65)		
Total	365 (20.10)	959 (52.80)	257 (14.20)	235 (12.9)		

Table 3 shows the demographic characteristics correlates of childhood experiences when categorized into the above 4 groups. Grades (*p* = 0.032) and sexual orientation (*p*< 0.01) are associated with childhood experiences.

### Association Between Childhood Experiences and Psychological Distress

Bivariate associations between childhood experience (e.g., Low-Both, High-BCEs, High-ACEs, and High-Both) and three psychological distress indicators (i.e., uncertainty stress, depressive symptoms, and suicidal ideation) are shown in Table 4. Results indicated that the childhood experiences are associated with all three psychological distress indicators (all *p*-value < 0.01). Notably, 27.53, 32.98, and 9.14% of participants reported uncertainty stress, depressive symptoms, and suicidal ideation, respectively. In addition, the High-ACEs group has the highest incidence of all three psychological distress indicators, while the High-BCEs group showed the lowest incidence of all three psychological distress indicators.

**TABLE 4:** Childhood experiences correlates of psychological distress.

Childhood experiences	Uncertainty stress*	Depressive symptoms*	Suicidal ideation*
	No. (%)	No. (%)	No. (%)
High-BCEs	165 (17.21)	173 (18.04)	32 (3.34)
High-Both	61 (25.96)	74 (31.49)	27 (11.49)
Low-Both	148 (40.55)	181 (49.59)	36 (9.86)
High-ACEs	126 (49.03)	171 (66.54)	71 (27.63)
Total	500 (27.53)	599 (32.98)	166 (9.14)

*p < 0.001 for association between childhood experiences and psychological distress correlate.

**FIGURE 1:**
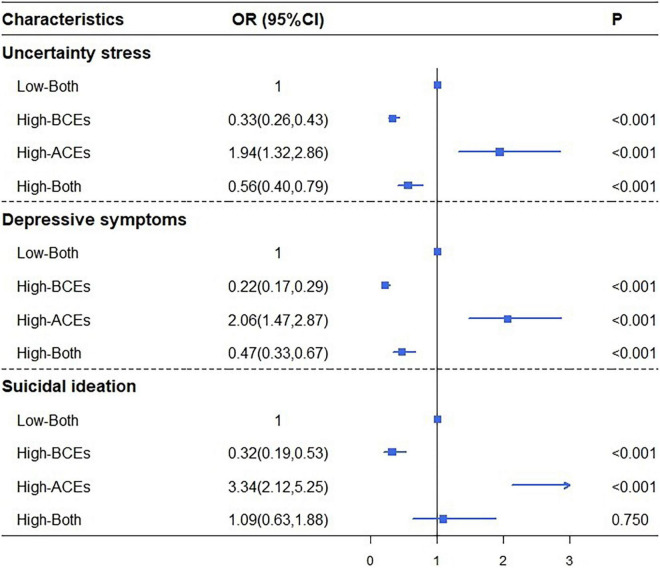
Associations between childhood experiences and psychological distress among Chinese undergraduates (referred to as Low-Both group). Covariates controlled in the logistic regression analysis were gender, grades, living expenses, only child, residence, and sexual orientation.

To better understand the relationship between the four groups of childhood experiences and psychological distress, we performed a logistic regression model, which used the Low-Both group as reference (Figure 1). High-BCEs group was less likely to report uncertainty stress [odds ratio (OR) = 0.33; 95%CI = 0.26, 0.43), depressive symptoms (OR = 0.22; 95%CI = 0.17, 0.29), and suicidal ideation (OR = 0.32; 95%CI = 0.19, 0.53) when compared with Low-Both group. Conversely, the High-ACEs group was about two times likely to report uncertainty stress (OR = 1.94; 95%CI = 1.32, 2.86) and depressive symptoms (OR = 2.06; 95%CI = 1.47, 2.87), and more than 3 times to have suicidal ideation (OR = 3.34; 95%CI = 2.25, 5.25). In addition, High-Both group had significant protective effects on uncertainty stress (OR = 0.56; 95%CI = 0.40, 0.79) and depressive symptoms (OR = 0.47; 95%CI = 0.33, 0.67) than Low-Both group. Furthermore, reduction in OR of High-Both group was found in all three psychological distress indicators (1.94 vs. 0.56; 2.06 vs. 0.47; 3.34 vs. 1.09) than High-ACEs group, and a loss of significance levels (0.00 vs. 0.75) of High-Both group was found in suicidal ideation. The logistic regression model that used the High-ACEs group or High-BCEs group as reference is shown in Supplementary Figures 1, 2. The association between childhood experiences and psychological distress by using continuous variables is shown in Supplementary Table 1. Results in Supplementary Table 1 revealed that BCEs have a significant protective effect in all three psychological distress indicators, while ACEs play as a risk factor in all three psychological distress indicators.

## Discussion

This study assessed the prevalence of ACEs, BCEs, and an overlap between them, as well as their association with psychological distress through a school-based investigation among undergraduates. All of the hypotheses were confirmed in this study. We have highlighted a substantially elevated risk of psychological distress among undergraduates with High-ACEs and a substantially reduced risk of psychological distress among undergraduates with High-BCEs. Moreover, BCEs could counteract the negative effects of ACEs on psychological distress, especially depressive symptoms and uncertainty stress.

In this study, about 27.1% of undergraduates experienced at least one type of ACEs, which is much lower than that of the adolescents in Brazil (85%) (Soares et al., 2016) and adults in the United States (82.9%) (Merrick et al., 2017) and China (66.2%) (Chang et al., 2019), but is similar to college students in China (35.16%) (Cheung et al., 2021). As discussed in the previous study (Butchart et al., 2006), there is a great deal of uncertainty around the estimates of the frequency and severity of ACEs worldwide due to inconsistencies in the measurement and definition of ACEs. In addition, it is worth noting that most previous studies assessed ACEs in the elder population (McLaughlin et al., 2010; Merrick et al., 2017), while few studies were conducted in young adults (Jia et al., 2020; Cheung et al., 2021). Much violence against children remains largely hidden and unreported because of fear and stigma and the societal acceptance of this type of violence (Pinheiro, 2006; Norman et al., 2012). Especially in Chinese undergraduates, Confucianism values and traditional cultures are rooted in thoughts (Ng, 1997), which may contribute to stigma formation, F., more shame toward ACEs (Mukolo et al., 2010; Purtle et al., 2021). Regarding BCEs, 65.7% of undergraduates reported more than 8 BCEs. Undergraduates reported higher BCEs in each item than homeless parents and pregnant women (Narayan et al., 2018; Merrick J.S. et al., 2019). The mean score (8.67 ± 1.78) of BCEs in this study is consistent with American adult (8.70 ± 1.68) and Chinese community people (8.63 ± 1.73) (Doom et al., 2021), and higher than the trauma-exposed sample (6.39 ± 2.66) (Karatzias et al., 2020).

Our findings suggested that BCEs were significantly correlated with lower psychological distress, while ACEs were significantly correlated with higher psychological distress. These findings verified our hypotheses 1 and 2. Similar results were found in other studies that both of them could predict the psychological distress independently (Narayan et al., 2018; Atzl et al., 2019; Bethell et al., 2019; Crandall et al., 2019; Merrick M.T. et al., 2019; Petruccelli et al., 2019; Doom et al., 2021). It is well known that ACEs are disadvantaged in the formation of secure attachment (Hill et al., 1994). In line with attachment theory, it is illustrated that secure attachment has a protective effect on psycho-social development of the children (Bretherton, 1985). Secure attachment is the major psychological resource of the children when suffering from troubles or frustrations (Violato and Arato, 2004). Therefore, children with ACEs might show less secure attachment and greater vulnerability in response to mental problems than those who experienced no obvious adversity (You et al., 2014).

In addition, the Resiliency Theory provides a theoretical framework for the effect of BCEs. Resilience includes the ability to cope when facing adversity (Cicchetti, 2016). BCEs reflect the internal and interpersonal resources from childhood (e.g., feeling comfortable with oneself, feeling safe with and close to others) which are often mentioned as aspects of resiliency and positive youth development (Klohnen, 1996; Masten et al., 1999; Skodol et al., 2007). Building BCEs for children at present could enhance their resilience to psychological distress in adulthood (Doom et al., 2021). Psychological distress could be buffered from pre-existing BCEs, resilience, and resources (Masten and Motti-Stefanidi, 2020).

Finally, as shown in Figure 1, the co-occurrence of ACEs and BCEs (High-Both group) has a protective effect on moderate psychological distress (uncertainty stress, depressive symptoms), which suggested that the positive effect of BCEs could not only counteract the negative effect of ACEs but also even reverse the negative effect of ACEs. These findings further confirmed our hypothesis 3. This can be explained by the effects of resilience, which could moderate the association between ACEs and mental problems (Poole et al., 2017). BCEs could largely counteract the negative effects of ACEs through enhancing resilience (Hornor, 2017; Poole et al., 2017; Doom et al., 2021) when suffering from moderate mental problems (uncertainty stress, depressive symptoms).

However, in suicidal ideation, the co-occurrence of ACEs and BCEs (the High-Both group) also showed a risk effect, which suggested that ACEs may have a more negative effect than the positive effect of BCEs. Although the High-Both group in Figure 1 is insignificant, the negative effect of ACEs was neutralized appropriately by BCEs. This can be explained by the challenge model (Zimmerman, 2013; Crandall et al., 2019), which posits that if the adversity is too great, then it will overwhelm the system and inhibits coping. When suffering from excessive adversities, moderate psychological distress will transfer to suicidal ideation, which is known as a serious public health problem (Bachmann, 2018). The positive effect of BCEs is weaker than the negative effect of ACEs when they co-occurred in such strong psychological distress (Felitti et al., 1998). These findings highlighted the urgent need to advocate BCEs in childhood, especially in moderate psychological distress, as well as a great need for the prevention of ACEs.

Unfortunately, events that have already occurred cannot be erased. Early screening of those at risk of ACEs and the development of effective interventions in early childhood should be encouraged to protect children from ACEs (Norman et al., 2012). It is most important to carry out widespread screening of BCEs in clinical settings, which would be beneficial for identifying those who may be at risk for current or future mental health problems (Narayan et al., 2021). Parents also play a role in shaping positive peer relationships or school and neighborhood environments to increase BCEs (Doom et al., 2021).

## Limitation

Several limitations should be noted in this study. First, we cannot attribute causality to the relationship between childhood experiences and distress due to the cross-sectional nature. Second, study data may be biased due to the entire self-reported questionnaire. Third, because participants were mostly collected from only three provinces of China and consisted of college students, the representativeness of the sample may be limited. As can be seen in this study, resilience has a close relationship with childhood experiences. In the future study, we would examine the role of resilience (Norman et al., 2012; Kumari, 2020) and gender (Al Shawi et al., 2019) in the association between childhood experiences and psychological distress based on a structural equation model to further explore the relationship between these factors. Future studies should also be conducted in broader populations to explore the effects of co-occurrence of ACEs and BCEs.

## Conclusion

Our findings suggested that ACEs and BCEs could not only predict the psychological distress independently, but also BCEs could counteract the negative effect of ACEs in psychological problems. These findings pointed out an even greater need to identify and support the victims of ACEs and an urgent need to increase BCEs in early childhood.

## Data Availability Statement

The raw data supporting the conclusions of this article will be made available by the authors, without undue reservation.

## Ethics Statement

The studies involving human participants were reviewed and approved by the Medical Ethics Committee of Xuzhou Medical University. The patients/participants provided their written informed consent to participate in this study.

## Author Contributions

HH contributed to data curation, formal analysis, investigation, methodology, project administration, resources, software, visualization, writing—original draft, reviewing, and editing. WW contributed to formal analysis, investigation, methodology, validation, writing, reviewing, editing, and funding acquisition. CZ, JT, JX, and JW contributed to validation, writing, reviewing, and editing. QZ, WY, and XG contributed to conceptualization, funding acquisition, supervision, writing, reviewing, and editing. All authors contributed to the article and approved the submitted version.

## Conflict of Interest

The authors declare that the research was conducted in the absence of any commercial or financial relationships that could be construed as a potential conflict of interest.

## Publisher’s Note

All claims expressed in this article are solely those of the authors and do not necessarily represent those of their affiliated organizations, or those of the publisher, the editors and the reviewers. Any product that may be evaluated in this article, or claim that may be made by its manufacturer, is not guaranteed or endorsed by the publisher.
