# Supplementary material for: Childhood Experiences and Psychological Distress: Can Benevolent Childhood Experiences Counteract the Negative Effects of Adverse Childhood Experiences?
Source: Front Psychol. 2022 Feb 25;13:800871. doi: 10.3389/fpsyg.2022.800871 (PMC8914177; doi:10.3389/fpsyg.2022.800871)
Supplement: Supplementary file 1 [file Data_Sheet_1.docx]

Supplementary Material

## Supplementary Figures


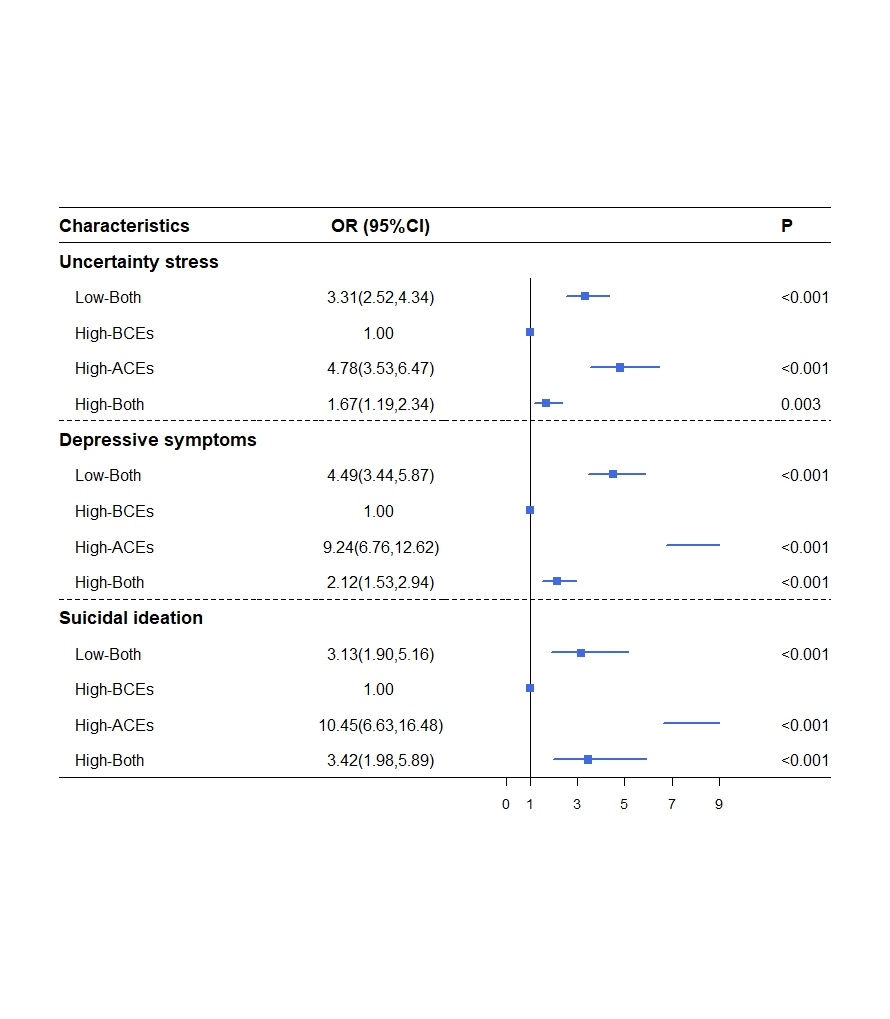


**Figure. S1** Associations between childhood experiences and psychological distress among Chinese undergraduates (reference as High-BCEs group)

Note: Covariates controlled in the logistic regression analysis were gender, grades, living expenses, only-child, residence, sexual orientation.


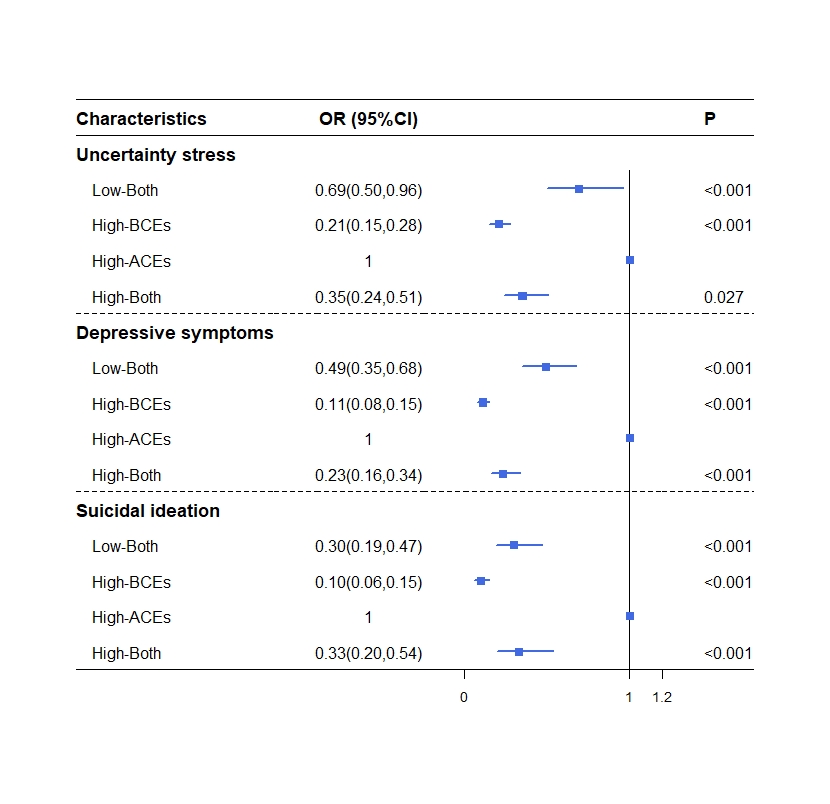


**Figure. S2** Associations between childhood experiences and psychological distress among Chinese undergraduates (reference as High-ACEs group)

Note: Covariates controlled in the logistic regression analysis were gender, grades, living expenses, only-child, residence, sexual orientation.

## Supplementary Tables

**Table S1.** Association between childhood experiences and Psychosocial distress among Chinese undergraduates.

|  | **R** | **R^2^** | **F** | ***P*** |  |  |  |
| --- | --- | --- | --- | --- | --- | --- | --- |
| **Uncertainty stress** | 0.411 | 0.169 | 45.785 | <0.001 | **Beta** | **t** | ***P*** |
| ACEs |  |  |  |  | 0.056 | 2.536 | 0.011 |
| BCEs |  |  |  |  | -0.387 | -17.492 | <0.001 |
| **Depressive symptoms** | 0.518 | 0.268 | 82.827 | <0.001 | **Beta** | **t** | ***P*** |
| ACEs |  |  |  |  | 0.123 | 5.936 | <0.001 |
| BCEs |  |  |  |  | -0.474 | -22.822 | <0.001 |
| **Suicidal ideation** | 0.329 | 0.108 | 27.420 | <0.001 | **Beta** | **t** | ***P*** |
| ACEs |  |  |  |  | 0.189 | 8.251 | <0.001 |
| BCEs |  |  |  |  | -0.191 | -8.327 | <0.001 |

Note: (1) Association between childhood experiences and uncertainty stress/ depression/suicidal ideation were used multiple liner regression respectively. (2) Covariates controlled in the regression analysis were gender, grades, living expenses, only-child, residence, sexual orientation.
